# First record of *Fusarium concentricum* (Hypocreales: Hypocreaceae) isolated from the moth *Polychrosis cunninhamiacola* (Lepidoptera: Tortricidae) as an entomopathogenic fungus

**DOI:** 10.1093/jisesa/iead008

**Published:** 2023-03-14

**Authors:** Hua-Long Qiu, Eduardo G P Fox, Chang-Sheng Qin, Hua Yang, Long-Yan Tian, De-Sen Wang, Jin-Zhu Xu

**Affiliations:** Guangdong Provincial Key Laboratory of Silviculture, Protection and Utilization/Guangdong Academy of Forestry, Guangzhou 510520, China; Programa de Pós-Graduação em Ambiente e Sociedade (PPGAS), Universidade Estadual de Goiás (UEG), Quirinópolis, Goiás 75860-000, Brazil; Guangdong Provincial Key Laboratory of Silviculture, Protection and Utilization/Guangdong Academy of Forestry, Guangzhou 510520, China; Guangdong Provincial Key Laboratory of Silviculture, Protection and Utilization/Guangdong Academy of Forestry, Guangzhou 510520, China; Guangdong Provincial Key Laboratory of Silviculture, Protection and Utilization/Guangdong Academy of Forestry, Guangzhou 510520, China; Department of Entomology, South China Agricultural University, Guangzhou 510642, China; Guangdong Provincial Key Laboratory of Silviculture, Protection and Utilization/Guangdong Academy of Forestry, Guangzhou 510520, China

**Keywords:** IPM, natural enemy, sustainable agriculture, entomopathogen

## Abstract

*Fusarium concentricum* Nirenberg & O’ Donnell (Ascomycota: Hypocreales) is a fungal species known to infect plants, but never reported as entomopathogenic. *Polychrosis cunninhamiacola* Liu et Pei (Lepidoptera: Tortricidae: Olethreutinae) is a major and widespread insect pest causing economic losses to cultivated Chinese fir *Cunninghamia lanceolata* (Lamb.) Hook. It is routinely controlled by extensive use of chemical insecticides, which is perceived as environmentally unsustainable. During March and April of 2019–2020, muscardine cadavers of larvae and pupae of *P. cunninhamiacola* infected with growing fungus were collected in a fir forest in northern Guangdong Province, China. Conidia were isolated and cultured on PDA medium, from which the fungal strain was identified as *F. concentricum* FCPC-L01 by morphology and by sequence alignment match with Tef-1α gene. Pathogenicity bioassays at the conidial concentration 1 × 10^7^ revealed *P. cunninhamiacola* adults and *Danaus chrysippus* (L.) (Lepidoptera: Nymphalidae) larvae are sensitive to the fungal infection, but not the fire ant *Solenopsis invicta* Buren (Hymenoptera: Formicidae). We believe results indicate this fungal strain might be applicable against specific target insect pests. As this is the first record of a natural infection caused by *F. concentricum* in insects, we propose host specificity tests should be done to evaluate its potential as a biocontrol agent.

## Introduction

**Fig. 1.**
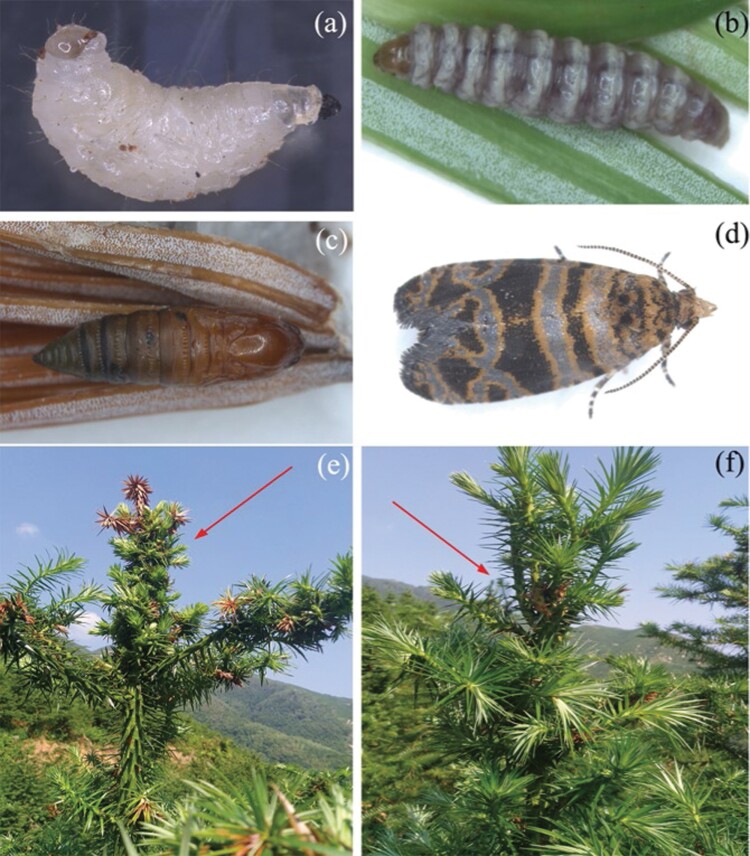
The life cycle of *Polychrosis cunninhamiacola* and its effects on the host plant. (a) Early instar larva， （b） late instar larva， （c）pupa， （d）adult， （e–f） attacked fir branch producing multiple shoots at lower height indicated with arrows. Unharmed trees usually present only one shoot at lower height for each tree; the onset of several shoots after attack by *P. cunninhamiacola* larvae will significantly affect tree growth.

The Chinese fir *Cunninghamia lanceolata* (Lamb.) Hook is a tree endemic to China reputed for its pronounced tolerance to ambient poor soil stress, growth speed, and quality of wood (Rong-Li et al. 2014). It is one the fastest growing timber species across southern provinces in China (Zeng et al. 2006). Nonetheless, the overall wood quality and seed yield of *C. lanceolata* has been threatened due to an increase in the number of diseases and pests affecting this species (Yu 2000):to date, there are 7 different diseases and 5 species of pest insects reported in China (Tian et al. 2019). For instance, the moth *Polychrosis cunninhamiacola* (Lepidoptera: Tortricidae: Olethreutinae) inflicts major losses to growers of *C. lanceolata* in China, and this pest has spread into southern provinces of China during the latest years, reaching, for example, the city of Shaoguan in Guangdong province (Liu et al. 2007, Peng et al. 2021). This moth is widespread across the *C. lanceolata*-producing provinces of Guangdong, Guangxi, Fujian, Jiangxi, Zhejiang, Guizhou, and Sichuan. Females of *P. cunninhamiacola* lay their eggs on the leaves of *C. lanceolata* and the hatching larvae travel to the top buds of young shoots, causing deleterious effects and marked malformations such as multiple apical shoots at lower height and trunk bending (Liu et al. 2007), also seriously impairing growth speed and wood quality (Fig. 1). Copious amounts of chemical pesticides are routinely used in attempting to control this insect pest, however since the planted areas of *C. lanceolata* are so vast, the practice has become also a significant concern because of the generated pollution that can severely affect the environment, the safety of forest-derived products, and kill natural enemies such as parasitic wasps (Hamburg and Guest 1997). Therefore, novel pest control strategies are now a necessity for managing forests composed of *C. lanceolata*.

Entomopathogenic fungi can infect and kill destructive insects, and thus play a central role in Integrated Pest Management (IPM) by offering a more natural protection through natural enemies, leading towards an ecological balance (Gul et al. 2014, Kidanud 2020). The fungal genera *Beauveria* and *Metarhizium* are two important entomopathogenic agents employed in controlling agricultural and forestry pest insects because of their wide host ranges (Meyling and Eilenberg 2007). Also, some strains of the genus *Fusarium* have been reported as efficient in controlling pest insects, exhibiting some of the characteristics desirable for an agent of agricultural and forestry biological control, such as delivering high mortality rates and presenting abundant sporulation capacity. *Fusarium* fungal strains are badly reputed as some species are considered plant pathogens and producers of mycotoxins (Mohanty et al. 2008, Munshi et al., 2008, Pelizza et al. 2011). To date, nine species of *Fusarium* have been demonstrated to be pathogenic to insects, attacking hosts within Lepidoptera, Diptera, Coleoptera, Hemiptera, Hymenoptera, and Orthoptera (Santos et al. 2020).

In this study, we isolated *F. concentricum* from dead moths and tested its toxicity against living *P. cunninhamiacola* and the other pest species *Danaus chrysippus* L.(Lepidoptera), and *Solenopsis invicta* Buren (Hymenoptera). The species *F. concentricum* belongs to the *F. fujikuroi* species complex, and is the most common fungus causing the fruit blotch in the roselle *Hibiscus sabdariffa*, and the pepper fruit rot in *Capsicum annuum* (Wang et al. 2013, Rahim et al. 2020). This species has never been recorded attacking insects. This research attempted to evaluate the potential for development and utilization of *F. concentricum* as an alternative biological agent for pest management.

## Materials and Methods

### Cadaver Collection and Species Identification of Fusarium Strain FCPC-L01

**Fig. 2.**
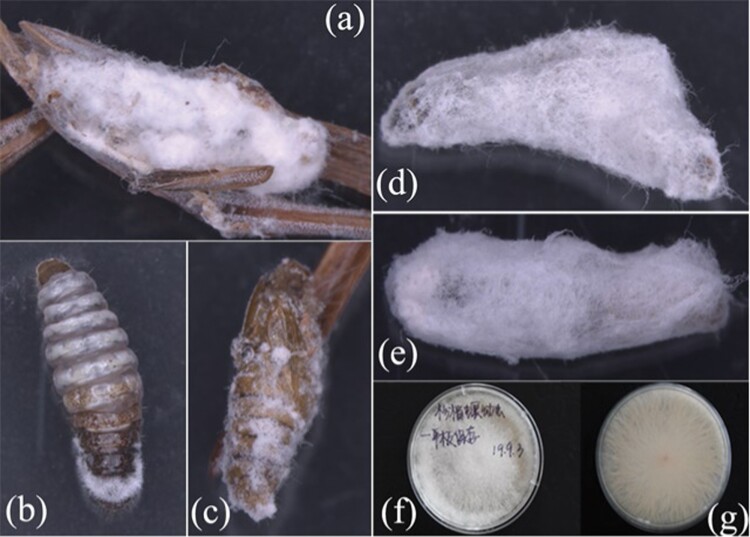
Fungus-infected dead larvae and pupae of *Polychrosis cunninhamiacola* (a–e) and frontal and rear view of a Petri dish containing entomopathogenic fungi isolated from the dead insects (f–g).

During the months of March and April of 2019 and 2020, the amount of damage inflicted by pest insects to a seed garden of *C. lanceolata* was evaluated in the northern part of the city of Shaoguan, Guangdong province of China (coordinates N:24.705, E:113.825). A large number of dead larvae and pupae of *P. cunninhamiacola* showing signs of fungal infection and sprouting white hyphae were found (Fig. 2). Careful inspection of conidial morphology under the microscope indicated that the growing fungi were neither *Beauveria bassiana* nor *Metarhizium anisopliae,* based on the presence of crescent shaped macro- and micro-conidia.

The conidia on the surface of cadavers were carefully sampled with a thin wire needle and seeded into a 2 ml tube containing 0.05 % Tween-80 solution, from which 0.1 ml – aliquots were spread onto Potato-Dextrose Agar medium (PDA, 200 g/L potato, 20 g/L dextrose, and 20 g/L agar) in 9-cm wide Petri dishes using a triangular glass rod (Du et al. 2019). The inoculated Petri dishes were incubated at 25 ± 2℃, relative humidity 75 ± 5% for 3–6 days. Sprouting hyphae were collected from a single colony in the plate, subcultured in a new PDA plate to produce a pure culture, and incubated for 8 days.

The fungi was further identified as genus *Fusarium* based on the elongation factor 1α (TEF-1α) (Kristernsen et al. 2005). DNA from *Fusarium* strain FCPC-L01 (isolated from larvae of *P. cunninhamiacola*) was extracted using a genomic DNA extraction kit (Axygen Biotechnology, Hangzhou, China). TEF-1α sequences of *Fusarium* strains were amplified by PCR using a set of primers (from Du et al. 2019): TEF-1αF:5ʹ-ATGGGTAAGGAGGACAAGAC-3ʹ and TEF-1αR:5ʹ-GGAAGTACCAGTGATCATGTT-3ʹ. The amplified product was sequenced by the Sanger method at Shenggong, Ltd, Gaugnzhou, China. The cloned TEF-1α gene sequences were aligned using ClustalW with MEGA5 software (Tamura et al. 2011). Gene homology was assessed using NCBI BLASTn queries and a phylogenetic tree constructed by Neighbor-Joining method using MEGA5 software with *Fusarium* FCPC-L01 and other model *Fusarium* strains and the outgroup *M. anisopliae*. The sequence was submitted to the GenBank and accession number (Genbank accession OP617256) was obtained.

### Preparation of Conidial Suspension

The *Fusarium* strain FCPC-L01 was maintained in Petri dishes containing PDA. Dishes were incubated at the constant temperature of 25 ± 1°C, 85 ± 1% RH for 8 days in an incubator. Conidia were carefully sampled with a sterilized brush and suspended in sterilized 0.05% Tween-80 water solution. The conidial concentration was measured using a haemocytometer, and adjusted to 1 × 10^7^ conidia/ml for further experiments.

### Pathogenicity Bioassay

Adults of *P. cunninhamiacola* were obtained from the *C. lanceolata* seed garden of Shaoguan city, larvae of *D. chrysippus* were also collected locally, nearby a seed garden, and workers of the fire ant *Solenopsis invicta* were collected from a public park in Guangzhou, China. All sampled insects were contained in plastic boxes (50 cm × 40 cm × 15 cm) placed in the dark in a thermal incubator set to 25 ± 1°C and 85 ± 1%relative humidity (RH). The fire ants were fed ad libitum with *Tenebrio molitor* larvae and 25% sucrose water, the lepidopteran larvae were fed leaves of their host plants and the adults fed 25% honey every other day.

For bioassays, samples of the different insects were placed in micro-centrifuge tubes containing conidial suspensions at the set concentration of 1 × 10^7^ conidia/ml. Specifically, for each species, a control group (*N* = 60) was treated with 0.05% Tween-80 aqueous solution. Per experimental groups, insects (*N* = 60) of each species were submerged into the conidial solution and gently swirled for five seconds, after which the excess liquid was removed from their surface by dabbing with a piece of ﬁlter paper for 10 min. The insects were then introduced into separated plastic boxes and kept in groups based on their treatments, and maintained under light: dark = 12h: 12h conditions at 25 ± 1°C and 85 ± 1% RH for 8 days. The mortality within each experimental group was recorded daily and the dead insects were removed to reduce the possibility of cross-contamination by *Fusarium* conidia. Removed cadavers were surface-sterilized and monitored for sprouting conidia for another 8 days.

### Plant Infection Bioassays

The *Fusarium* strains FCPC-L01, SM-LK-2, and SM-LK-b5 were selected for plant infection bioassays. SM-LK-2, and SM-LK-b5 were isolated from plants of *C. lanceolata.* Aqueous solution of 0.05% Tween-80 was used as control. Preparation of conidial suspensions of SM-LK-2 and SM-LK-b5a was the same as described above of FCPC-L01 for the treatment of the experimental insects. Leaves of healthy *C. lanceolata* seedlings (10-cm high) were sprayed with 2-mL conidial suspensions (10^7^ spore/ml) (*N* = 10) of one the different strains. The control was 0.05% Tween-80 aqueous (*N* = 10). The plants were then maintained under 12: 12 h dark: light conditions at 25 ± 1°C and 75 ± 1% RH. The morphological state of the plants such as the color of leaves and mycelium growth on the surface of leaves in each group were recorded daily for 7 days.

### Statistical Analysis

All collected data were analyzed using SPSS v.22.0. Mortality bioassays based on the median lethal time (LT_50_) were examined using Probit regression analysis. The survivorship distributions at different treatment groups were compared by the Breslow statistics (Kaplan-Meier survival test), and the hazard ratio of death was analyzed using the Cox Proportional Regression analysis to generate the Wald Statistic.

## Results

### Identification of *Fusarium* Strain FCPC-L01

**Fig. 3.**
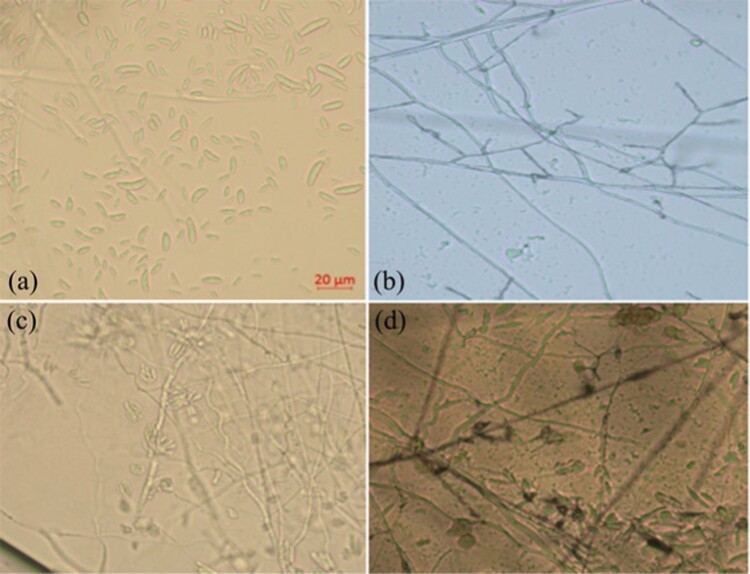
Morphology of conidia and mycelium of the fungal strain *Fusarium concentricum* FCPC_L01. (a) macroconidia and microconidia, (b–d) mycelium and conidiophores. Scale bars measured 20 μm for a–d.

The isolated fungus cultivated on PDA was morphologically identified as *Fusarium* sp. according to the conidial shape of two conidial forms: macroconidia and microconidia. Macroconidia grew pale-orange, slightly falcate, or else straight, sporodochia with thin walls and basal foot-shaped cells, bearing 3–4 septates measuring 1–2 × 13–17 μm (Fig. 3a). Microconidia were more abundant than macroconidia and oval in shape; microconidia appeared in chains on the monophialides, usually 0 septate and 1–2 × 3–8 μm (Fig. 3a–d). Such characteristics are typical of *Fusarium* spp.

**Fig. 4.**
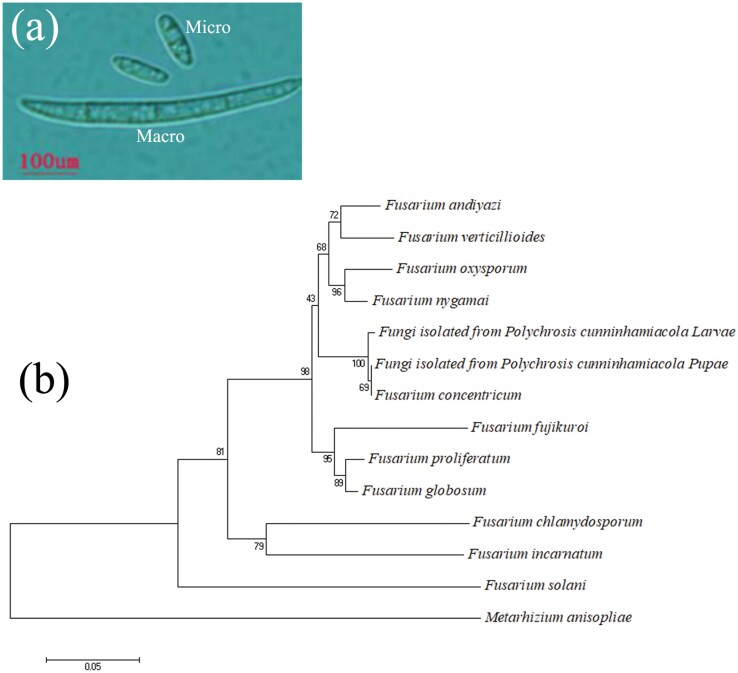
Characteristic macroconidia and microconidia of the fungal species (a) and neighbor joining phylogenetic tree plotted with MEGA 5 of entomopathogenic fungi relative to *Fusarium* species (b).

The TEF-1α sequence length of *Fusarium* FCPC-L01 was 678 bp (Fig. S1, GenBank accession number OP617256). Neighbor-Joining phylogenetic trees based on the rDNA ITS region sequences were constructed with bootstrap value of 1,000, indicating *Fusarium* FCPC-L01 is well supported within the *F. concentricum* clade (Fig. 4; Fig. S2). In all generated trees *Fusarium* FCPC-L01 (Genbank accession OP617256) fell within the same clade branch as *F. concentricum.*

### Pathogenicity Bioassay

**Fig. 5.**
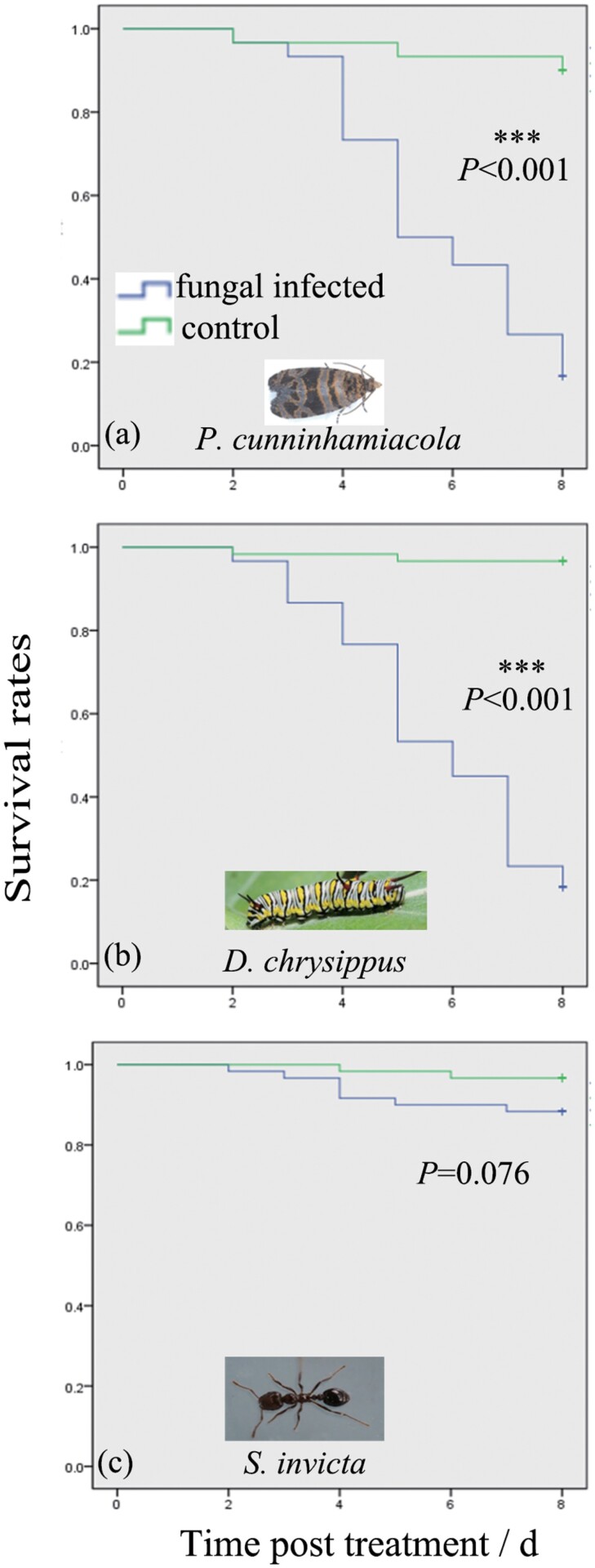
Toxicity bioassays from application of a fungal strain of *Fusarium concentricum* at the maximum possible dosage of 1 × 10^7^ conidia/ml against adult *Polychrosis cunninhamiacola* moths, *Danaus chrysippus* caterpillars, and *Solenopsis invicta* workers.

The bioassay data of the concentration-response pathogenicity against the pest insect species of *P. cunninhamiacola*, *D. chrysippus*, and *S. invicta* are shown in Fig. 5, with their respective Median lethal times (LT_50_) values presented in Table 1. The corrected mortalities of fungus-exposed *P. cunninhamiacola, D. chrysippus*, and *S. invicta* were 81.48%, 81.03%, and 8.62%, respectively (Fig. 5). The LT_50_ values of FCPC-L01 at the concentration of 1 × 10^7^ against *P. cunninhamiacola, D. chrysippus*, and *S. invicta* on the 8th day postinfection were respectively 5.94, 5.73, and 14.17 days (Table 1). Insects treated with *F. concentricum* at the concentration of 1 × 10^7^ conidia/ml presented different survivorship patterns: in *P. cunninhamiacola* and *D. chrysippus* fungal infection were significant predictors of mortality (*P. cunninhamiacola*: Wald Statistic = 18.29, df = 1, *P*< 0.001, Fig. 5a; *D. chrysippus*: Wald Statistic = 26.06, df = 1, *P*< 0.001, Fig. 5b), meaning fungal treatments delivered mortalities 14.02 and 40.30 times greater than their respective control groups. On the other hand, no significant difference was perceived in the survivorships of *S. invicta* under fungal and control treatments (Wald Statistic =2.61, df = 1, *P* = 0.076, Fig. 5c).

**Table 1.** Median lethal times (LT_50_) of the fungal strain *F. concentricum* FCPC-L01 at the concentration of 1 × 1 0^7^ conidia/ml against three pest insect species, on the 8th day postinfection

Insect species	LT_50_/d	95% Confidence interval	Regression equation^a^	χ^2^	*P* ^b^
* Polychrosis cunninhamiacola*	5.94	5.19–6.76	m = −0.53t −3.15	9.82	0.13
*Danaus chrysippus*	5.73	5.42–6.05	m = 0.48t − 2.76	7.35	0.29
*Solenopsis invicta*	14.17	10.75–31.73	m = 0.19t − 2.74	5.76	0.45

^a^ m = Probit transformed mortality; t = Time posttreatment.

^b^ Homogeneity for the fit was accepted if *P* > 0.05 for the χ^2^ test (df = 6).

Overall, 85% of the fungus-exposed dead lepidopterans developed *F. concentricum* hyphae (Fig. S3 in Supplementary files), but none within control groups, indicating fungal infection was likely the cause of death.

### Plant Infection Bioassay

**Fig. 6.**
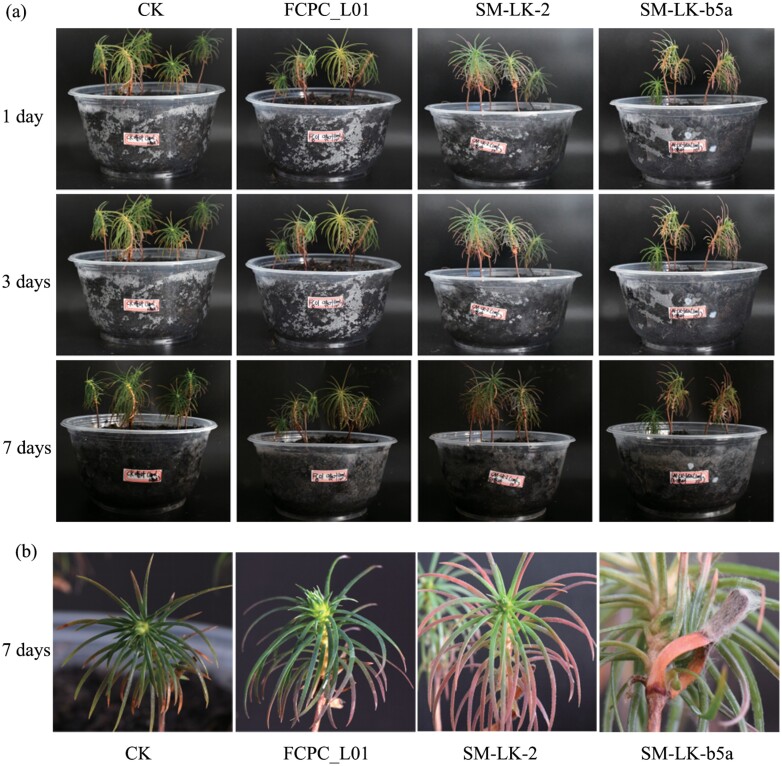
Susceptibility to infection by Chinese fir *Cunninghamia lanceolata* seedlings sprayed with conidial suspensions of three different species of *Fusarium* fungi. (a) Morphology of Chinese fir seedling at different days posttreatment, (b) at the 7th day posttreatment, CK in the graph represents control group.

The effects from exposure to *Fusarium* conidia on *C. lanceolata* are shown in Fig. 6. On the 7th day posttreatment with conidial suspensions of either FCPC-L01 or Tween-80 control, no disease symptoms were observed (Fig. 6a) nor mycelia were observed on leaves (Fig. 6b). On the other hand, SM-LK-b5a seedlings showed evident wilting and hyphae forming on the leaves surface (Fig. 6b). Regarding SM-LK-2, no mycelia were noted on leaf surfaces, while leaf chlorosis was evident.

## Discussion

Entomopathogenic fungi are important biological control agents for use in agricultural and forestry pest management. In this study, dead *P. cunninhamiacola* larvae and pupae infected with an entomopathogenic fungus in the field were collected, which was isolated and identified as *F. concentricum,* morphologically and molecularly. The obtained *F. concentricum* strain proved pathogenic against adults of *P. cunninhamiacola* and larvae of *D. chrysippus*, but showed low toxicity against *S. invicta* workers in the laboratory. As far as we know, this is the first record of natural infection by *F. concentricum* in insects.

Correct species identification of *Fusarium* samples can be challenging based on morphology alone (Santos et al. 2020), as the approach grossly underestimates the extreme diversity of species in this genus. Therefore, molecular phylogeny is advisable to enhance accurate identification of *Fusarium* species (Chandra et al. 2011). The choice for rDNA internal transcribed spacer (ITS) sequence polymorphisms is often used for fungus identification, particularly in the *Metarhizium* genus (Nishi et al. 2011), however, as there is considerably less ITS polymorphism in *Fusarium* (Chandra et al. 2011) the gene TEF-1α was chosen because it contains enough polymorphism for species identification in *Fusarium* (Divakara et al., 2014).

Previous proposals for using *Fusarium* spp. as biological control agents have been largely dismissed, given the fact that numerous species produce harmful mycotoxins such as deoxynivalenol, trichothecenes, moniliformin, and zearalenone, potentially harmful to humans and the environment (Mello et al. 1999). Notwithstanding, it is also a fact that the technical understanding of the mycotoxins produced by commercially-available fungal biological control agents is scant, which currently include mycoinsecticides, mycoherbicides, and mycoparasites. In addition, a large body of knowledge regarding mycotoxins produced by *Fusarium* now exists, which should facilitate the selection of benign *Fusarium* strains by surveying the presence of mycotoxins through high sensitivity detection methods such as GC-MS and LC-MS (Stpień et al. 2019).

In order to sustain responsible use of insect-pathogenic fungi like *Fusarium* as biological control agents, it is also important to keep track of both target and non-target organisms being affected by each application system and context. For example, *Fusarium oxysporum* isolated from the brown planthopper *Nilaparvata lugens* showed no infection capacity against the host plants rice, cotton, or tomato, which is an indication that *F. oxysporum* strain should be safe for the host plants but infective against *N. lugens* (Kolander 2010). Similarly, the use of a saprophyte *Fusarium* species resulted in a reduction in the numbers of eggs, larvae, and adults of the pest bug *Daktulosphaira vitifoliae* (Hemiptera: Phylloxeridae) on grapes, but did not produce any infections on the host plant (Morquer and Nysterakis 1944). However, some strains of *F. oxysporum* isolated from *Sitona hispidula* (Coleoptera: Curculionidae) proved not only pathogenic to the insects’ larvae, but also to the ornamental clover *Trifolium pretense* (Kilpatrick 1961). In the present study, FCPC-L01 strain delivered high mortalities to two species of lepidopteran pests. However, the strain did not infect any branches or leaves of the host plant fir *C. lanceolata*. FCPC-L01 infection resulted in low susceptibility against *S. invicta*, which suggest FCPC-L01 might pose low risk of infection against ants in forested areas. It should be emphasized that such susceptibility tests were conducted under optimal conditions for fungal growth. Further bioassays are needed against a wider range of insect species which are known to exist in the fir field where this strain was obtained, in order to provide a more realistic preview of expectable outcomes on non-target insects.

In conclusion, this study was the first to record *F. concentricum* attacking *P. cunninhamiacola* moths, extending the body of knowledge about the pathogenic capacity of this *Fusarium* species against lepidopterans. Future studies could evaluate mycotoxin production against nontarget insects and associated potential effects on plants and humans, as to expand our understanding of the potential to exploit host-*Fusarium*-environment interactions.

## Supplementary Material

iead008_suppl_Supplementary_Material
