# Supplementary material for: First record of Fusarium concentricum (Hypocreales: Hypocreaceae) isolated from the moth Polychrosis cunninhamiacola (Lepidoptera: Tortricidae) as an entomopathogenic fungus
Source: J Insect Sci. 2023 Mar 14;23(2):2. doi: 10.1093/jisesa/iead008 (PMC10011878; doi:10.1093/jisesa/iead008)
Supplement: iead008_suppl_Supplementary_Material [file iead008_suppl_supplementary_material.docx]

**Supplementary file**

ccttataccg ttcgtcatcg ggcacgtcga ctctggcaag tcgaccactg tgagtactac 60

ccttgacgat gagcttatct gccatcgtaa tcctgaccaa gatctggcgg ggtgtatctc 120

aaaagacaac atgctgacat agcttcacag accggtcact tgatctacca gtgcggtggt 180

atcgacaagc gaaccatcga gaagttcgag aaggttagtc tcccttcgag cgcgcgtcct 240

ttgcccatcg attttcccct acgactcgaa acgtgcccgc taccccgctc gagaccaaaa 300

attttgcgat atgaccgtaa ttttttttgg tggggcattt accccgccac tcgagcgatg 360

ggcgcgtttg ccctctccac aatctcaatg agcgcatcgt cacgtgccaa gcagtcacta 420

accatccgac aataggaagc cgctgagctc ggtaagggtt ccttcaagta cgcctgggtt 480

cttgacaagc tcaaggccga gcgtgagcgt ggtatcacca tcgatatcgc tctctggaag 540

ttcgagactc ctcgctacta tgtcaccgtc attggtatgt tgtcgcctat gcttcattct 600

tcttcctcgt actaacatat cactcagacg ctcccggtca ccgtgatttc atcaagaaca 660

tgatcactgg tacttcca 678

**Fig. S1 Partial base sequence of TEF-1α of FCPC-L01 strain.**

1.


**Fig. S2.** Phylogenetic trees of *Fusarium* spp. using TEF-1α gene by minimum evolution (a) and maximum likelihood (b) methods


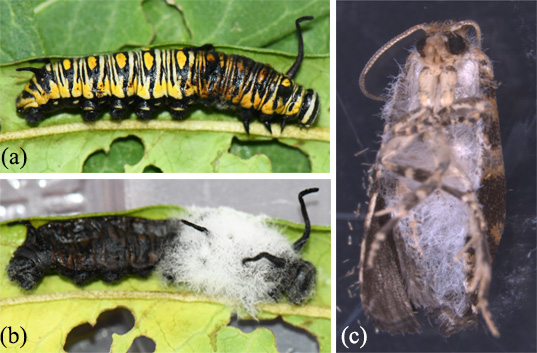


**Fig. S3.** Hyphae and conidia of *Fusarium concentricum* sprouting from the cuticle of infected lepidopteran insects. (a) Melanization of *Danaus chrysippus* caterpillar exposed to *F. concentricum* conidia*,* and (b) hyphae and conidia sprouting from the surface of a dead caterpillar; (c) hyphae and conidia sprouting from the abdomen of *Polychrosis cunninhamiacola* adults.
